# Nuclear factor erythroid 2-related factor 2 activation in streptozotocin-induced diabetic rats normalize renal hemodynamics and oxygen consumption

**DOI:** 10.48101/ujms.v129.10791

**Published:** 2024-07-09

**Authors:** Patrik Persson

**Affiliations:** a Division of Integrative Physiology, Department of Medical Cell Biology, Uppsala University, Uppsala, Sweden; b Department of Animal Biosciences, Swedish University of Agricultural Sciences, Uppsala, Sweden

**Keywords:** Glomerular filtration rate, renal blood flow, filtration fraction, mitochondria, DL-sulforaphane

## Abstract

**Background:**

Diabetic kidney disease is a major contributor to end stage renal disease. A change in kidney oxygen homeostasis leading to decreased tissue oxygen tension is an important factor initiating alterations in kidney function in diabetes. However, the mechanism contributing to changed oxygen homeostasis is still unclear. Hyperglycemia-induced production of reactive oxygen species and an altered response to them have previously been demonstrated. In the present study, chronic treatment with DL-sulforaphane to induce nuclear factor erythroid 2-related factor 2 (Nrf2) expression, a master transcriptional regulator binding to antioxidant response elements inducing increased protection against reactive oxygen species, is studied.

**Methods:**

Sprague–Dawley rats were made diabetic using streptozotocin and either left untreated or received daily subcutaneous injections of DL-sulforaphane for 4 weeks. Age-matched non-diabetic rats served as controls. After 4 weeks of treatment, rats were anesthetized using thiobutabarbital, and kidney functions were studied in terms of glomerular filtration rate (GFR), renal blood flow (RBF), sodium transport, kidney oxygen consumption, and kidney oxygen tension. Mitochondria was isolated from kidney cortical tissue and investigated using high-resolution respirometry.

**Results:**

GFR was increased in diabetics but not RBF resulting in increased filtration fraction in diabetics. DL-sulforaphane treatment did not affect RBF and GFR in controls but decreased the same parameters in diabetics. Increased GFR resulted in increased sodium transport and oxygen consumption, hence decreased efficiency in diabetics compared to controls. Increased oxygen consumption in diabetics resulted in decreased cortical tissue oxygen tension. DL-sulforaphane treatment decreased oxygen consumption in diabetics, whereas transport efficiency was not significantly affected. DL-sulforaphane treatment increased cortical pO_2_ in diabetics.

**Conclusions:**

DL-sulforaphane treatment affects renal hemodynamics, improving cortical oxygen tension but not mitochondrial efficiency.

## Introduction

Diabetic kidney disease (DKD) is a major contributor to end stage renal disease, requiring life-long renal replacement therapy. Pathological alterations early in the progression of DKD include changes to kidney oxygen metabolism, manifested as increased oxygen consumption not matched by increased oxygen delivery, ultimately causing a shortage of intra-renal oxygen availability (1, 2). Mechanistically, decreased oxygen availability is explained by decreased efficiency of adenosine triphosphate (ATP) production initiated by upregulation and activation of mitochondrial uncoupling (3, 4), a mechanism whereby mitochondria release protons independently of the ATP-synthase to alleviate mitochondrial membrane potential. Decreasing mitochondrial membrane potential decreases the probability for electrons in the electron transport chain reacting with oxygen at complex I or II, forming the oxygen radical superoxide. Hence, mitochondrial uncoupling is a mechanism allowing for dynamic regulation of mitochondrial superoxide production (5–7). In diabetes, mitochondrial uncoupling is increased, resulting in increased oxygen consumption for the same amount of ATP production (3). Due to renal autoregulation to maintain glomerular filtration rate (GFR), renal blood flow (RBF) cannot increase sufficiently to supply oxygen, resulting in a permanently decreased oxygen tension in the kidney (8). Treatment with coenzyme Q10, targeting oxygen radical formation at the electron transport chain, maintains mitochondrial coupling and efficiency in diabetes, linking oxygen radicals to decreased mitochondrial efficiency (4).

Nuclear factor erythroid 2-related factor 2 (Nrf2) is a transcription factor regulating genes involved in the degradation of reactive oxygen species such as the glutathione and thioredoxin antioxidant system, NADPH regeneration, and heme metabolism, thus providing protection against increased oxidative stress (9). Nrf2 is constitutively expressed, but its half-life is short due to degradation by proteasomes. This degradation is induced by binding to Kelch-like ECH-associated protein 1 (Keap1). Cellular stress responses induce Nrf2 by targeting Keap1 cysteine thiols, decreasing the ability for Keap1 to bind Nrf2 and subsequently decreasing its degradation (10). In the absence of Keap1 binding capacity, Nrf2 can translocate to the nucleus and induce transcription. Diabetes is closely connected to an increased load of oxidative stress, and the activation of the Nrf2 pathway has been considered a therapeutic target for diabetic complications (11). Sulforaphane is an isothiocyanate previously shown to induce Nrf2-related gene expression and offers protection in acute kidney injury models, such as ischemia-reperfusion injury and cisplatin-induced renal injury (12, 13). However, the regulation of kidney oxygen homeostasis in the streptozotocin-induced diabetes models after chronic sulforaphane treatment has not been investigated. We hypothesize that the activation of the Nrf2 pathway by sulforaphane will prevent changes in kidney oxygen homeostasis in diabetes.

## Materials and methods

### Animals and chronic treatment

Rats included in the study were purchased from Charles River (Italy) and housed in a temperature-controlled environment with a 12-h light cycle. Rats were included at 12 weeks of age, and diabetes was induced by a single tail vein injection of streptozotocin (50 mg/kg bw, Sigma-Aldrich, St. Louis, USA) dissolved in sterile 0.9% NaCl solution. Diabetes was confirmed 48 h post streptozotocin injection by a blood sample taken from the cut tip of the tail using a blood glucose monitoring device (Freestyle Lite, Abbott Diabetes Cara, Alameda, CA, USA). Blood glucose level >18 mmol/L was considered diabetic. Twenty rats were made diabetic, and 20 age-matched non-diabetic rats served as controls. Control and diabetic rats were divided into four groups, either receiving daily subcutaneous injection of DL-sulforaphane (0.5 mg/kg bw., Calbiochem) dissolved in DMSO and diluted in sterile 0.9% NaCl solution (DMSO concentration 1%) or vehicle. Treatment period was 28 days, after which the rats were analyzed for *in vivo* kidney function, mitochondria isolation for respirometry analysis, and tissue harvesting. All experiments were approved by the local committee for the care and use of research animals and in accordance with the EU directive on the protection of animals used for scientific purposes. The total number of rats included in the study was 40.

### In vivo experiments

On the day of experiments, rats were anesthetized with thiobutabarbital (Inactin, Sigma-Aldrich) at a dose of 80–120 mg/kg bw by intraperitoneal injection. The rats were placed on a servo-controlled heating pad maintaining a core body temperature at 37°C. Rats were tracheostomized to ensure spontaneous breathing, and catheters were placed in femoral artery and vein for blood pressure measurement, blood sampling, and infusion of Ringer’s solution (5 ml/kg/h for controls and 10 mL/kg/h for diabetics) containing ^3^H-inulin (American Radiolabeled Chemicals, St. Louis, MO, USA). The left kidney was exposed and immobilized in a plastic cup. The renal artery was dissected free, and an ultrasound probe was placed around it to allow for continuous RBF measurement. A catheter was placed in the ureter for continuous urine collection. The rats were allowed a recovery period before the experiments started. Urine was collected for a 30-min period, and an arterial blood sample was collected halfway through for the determination of plasma ^3^H-inulin concentration and arterial blood gas analysis. Renal vein samples were collected for blood gas analysis to be able to calculate total renal oxygen consumption. Renal tissue oxygen tension was measured by a Clark-type microelectrode mounted on a micromanipulator, allowing for precise measurements in the renal cortex at 1 mm depth from the kidney surface.

GFR was calculated by measuring urine flow rate (Vu) gravimetrically and urine and plasma ^3^H-inulin concentration by standard liquid scintillation technique. RBF was measured by an ultrasound flow probe (Transonic Systems, Itacha, NY, USA). Total renal oxygen consumption was measured by the difference in arterial and renal vein oxygen delivery obtained by blood gas analysis (iSTAT System, Abbot) and RBF. Cortical tissue oxygen tension was measured by Clark-type microelectrodes with a tip diameter of 10 µm (Unisense, Aarhus, Denmark). Electrodes were two-point calibrated in water equilibrated with air at 37°C (147 mmHg O_2_) and in a saturated solution of sodium sulfite (0 mmHg O_2_). Sodium and potassium in plasma and urine were determined by flame photometry (IL Instrument, Milan, Italy), and excretion rates were subsequently calculated.

### Mitochondria experiments

Renal cortical mitochondria were isolated by homogenization and differential centrifugation as previously described (14). In brief, kidney cortex was dissected on ice and homogenized in ice-cold isolation buffer (containing in mmol/L, 250 sucrose, 10 HEPES, 1 EGTA, and 1 g/L BSA at pH 7.4, 300 mOsm/kgH_2_O) by a Potter-Elvehjem glass homogenized rotating at 800 rpm. The homogenate was centrifuged at 800 g for 10 min, and supernatant was transferred to a new tube and centrifuged at 8,000 g for 10 min. The supernatant was discarded, and the pellet was suspended in mitochondria preservation media (containing 0.5 mmol/L EGTA, 3 mmol/L MgCl_2_, 60 mmol/L K-lactobionate, 20 mmol/L taurine, 10 mmol/L KH_2_PO_4_, 20 mmol/L HEPES, 110 mmol/L sucrose, 20 mmol/L histidine, 20 umol/L vitamin E succinate, 3 mmol/L glutathione, 1 umol/L leupeptine, 2 mmol/L glutamate, 2 mmol/L malate, 2 mmol/L Mg-ATP, and 1 g/L BSA essentially fatty-acid free). The volume of preservation media was calculated to 0.6 ul/mg initial sample weight. Samples were stored on ice until respiration experiments. Mitochondrial respiration was measured by high-resolution respirometry (Oxygraph 2k, Oroboros Instruments, Innsbruck, Austria) in respiration media (containing 0.5 mmol/L EGTA, 3 mmol/L MgCl_2_, 60 mmol/L K-lactobionate, 20 mmol/L taurine, 10 mmol/L KH_2_PO_4_, 20 mmol/L HEPES, 110 mmol/L sucrose, and 1 g/L BSA essentially fatty-acid free). Respiration was recorded during the presence of mitochondria, pyruvate (5 mmol/L), and malatate (2 mmol/L) (state 2). Maximal complex I-mediated respiration was recorded after the addition of adenosine diphosphate (ADP) (2.5 mmol/L) (state 3), followed by the addition of succinate (10 mmol/L) to reach maximal complex I + II-mediated respiration. Respiratory control ratio was determined by dividing state 3 respiration by state 2 respiration. To determine leak respiration, recording was performed after the addition of mitochondria, pyruvate, malate, and oligomycin (2.5 umol/L). Uncoupling protein (UCP)-mediated leak respiration was determined by the change in respiration after the addition of guanosine diphosphate (GDP) (2 mmol/L). Leak respiration mediated by the adenine nucleotide translocase (ANT) was determined by the change in respiration after the addition of carboxyactractyloside (5 umol/L). Mitochondria respiration was normalized to mitochondria protein concentration measured by DC Protein Assay (BioRad, Hercules, CA, USA).

### Statistical analysis

All statistical analysis was performed using GraphPad Prism (GraphPad software, SanDiego, CA, USA). Comparison between groups and treatment was performed using two-way analysis of variance (ANOVA), followed by Fisher’ LSD test. The test was two-tailed, and a *P*-value < 0.05 was considered statistically significant. All data are presented as the mean ± SEM.

## Results

**Figure 1:**
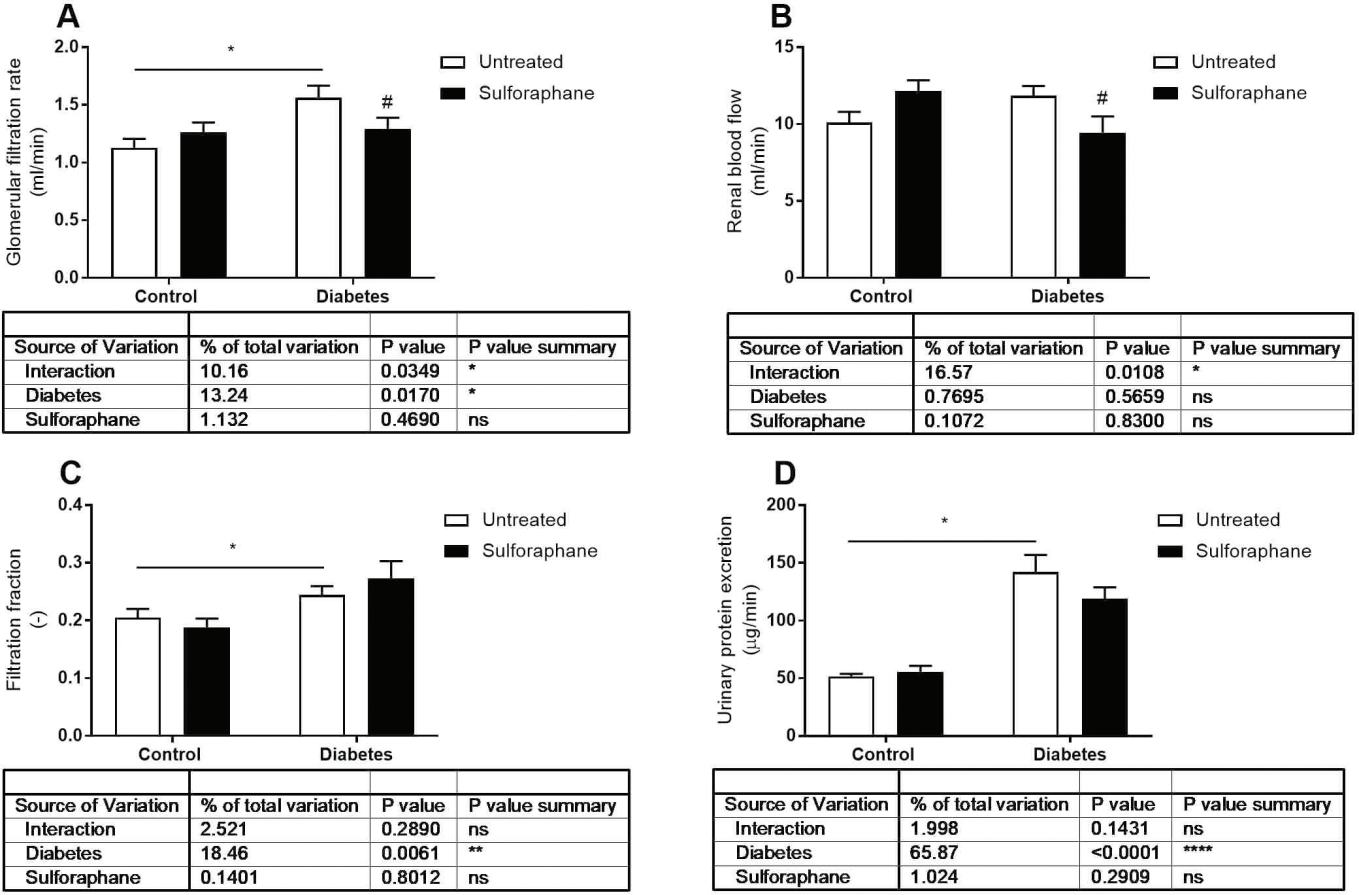
A: glomerular filtration rate (GFR), B: renal blood flow (RBF), C: filtration fraction (FF), and D: urinary protein excretion in control and streptozotocin-induced diabetic rats either untreated or after 4 weeks chronic DL-sulforaphane treatment. Untreated control *n* = 11, untreated diabetic *n* = 9, DL-sulforaphane treated control *n* = 9, and DL-sulforaphane treated diabetic *n* = 11. *P < 0.05 from two-way ANOVA, ^#^*P* < 0.05 compared to corresponding untreated group from Fisher’s LSD test.

**Figure 2:**
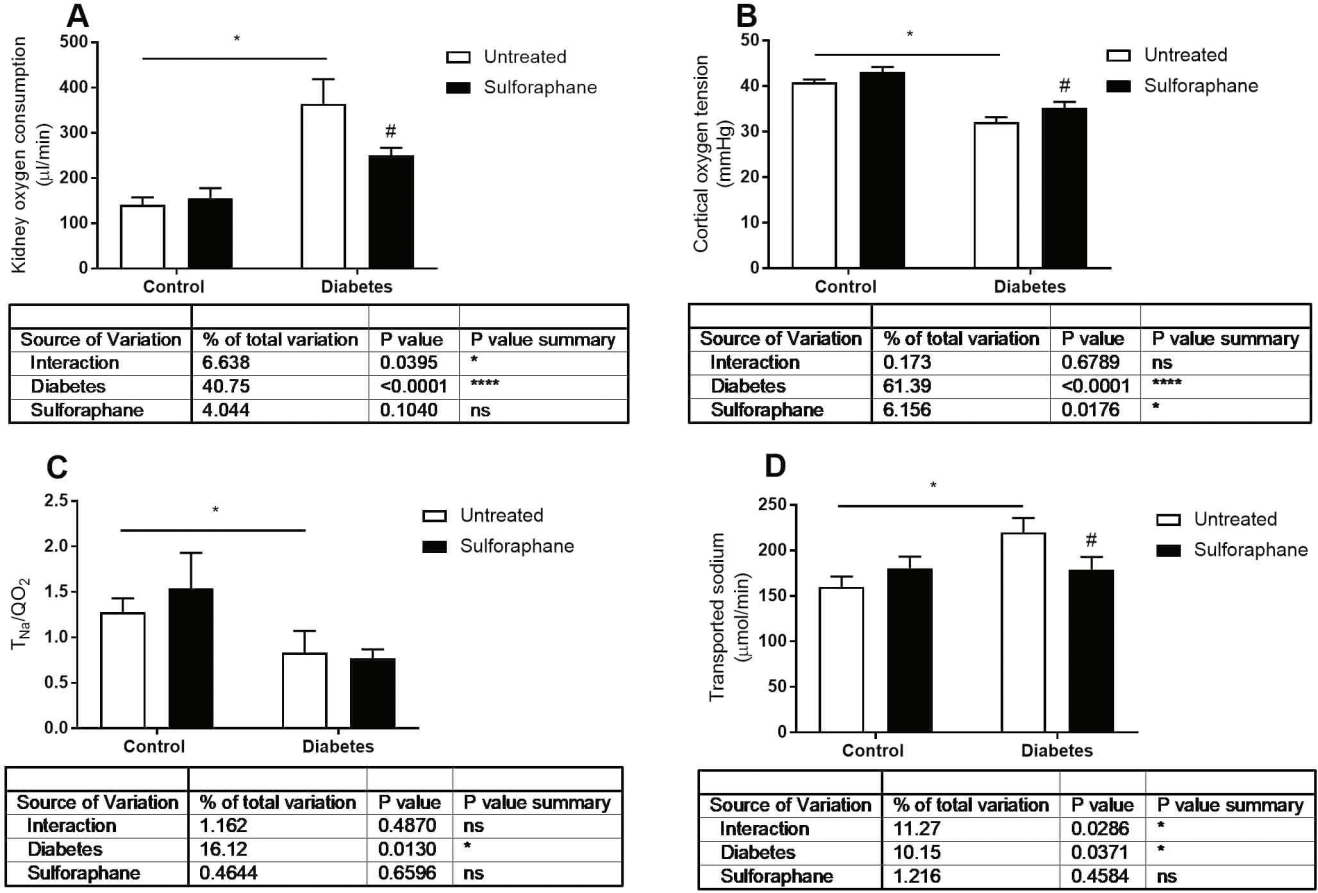
A: kidney oxygen consumption (QO_2_), B: cortical oxygen tension, C: transported sodium per consumed oxygen (T_Na_/QO_2_), and D: transported sodium (T_Na+_) in control and streptozotocin-induced diabetic rats either untreated or after 4 weeks chronic DL-sulforaphane treatment. Untreated control *n* = 11, untreated diabetic *n* = 9, DL-sulforaphane treated control *n* = 9, and DL-sulforaphane treated diabetic *n* = 11. *P < 0.05 from two-way ANOVA, ^#^*P* < 0.05 compared to corresponding untreated group from Fisher’s LSD test.

Blood glucose and left kidney weights were increased in diabetic rats compared to age-matched controls and were not affected by daily DL-sulforaphane treatment. Body weight was decreased in diabetics compared to age-matched controls and was also not affected by daily DL-sulforaphane treatment (Table 1). GFR was increased in diabetics, but not RBF resulting in an increased filtration fraction (FF) in diabetics. DL-sulforaphane treatment did not significantly affect RBF and GFR in controls but decreased RBF and GFR in diabetics (Figure 1). The increased GFR resulted in increased T_Na+_ and QO_2_, hence decreased T_Na+_/QO_2_ in diabetics compared to controls. Increased QO_2_ in diabetics also resulted in decreased cortical tissue pO_2_ (Figure 2). DL-sulforaphane treatment decreased QO_2_ in diabetics, whereas T_Na_+/QO_2_ was not significantly affected by DL-sulforaphane treatment. However, DL-sulforaphane treatment increased cortical pO_2_ in diabetics. Urinary protein excretion was increased in diabetics but was not significantly affected by DL-sulforaphane treatment. Mitochondrial function was not significantly different between controls and diabetics, and DL-sulforaphane treatment did not significantly affect mitochondrial function (Table 2).

**Table 1:** Body weight, blood glucose, left kidney weight, urinary flow and hematocrit (Hct) in untreated and DL-sulforaphane treated control and streptozotocin-diabetic rats.

Groups	Treatment	Body weight (g)	Blood glucose (mmol/L)	Left kidney weight (g)	Urinary flow (µL/min/kidney)	Hct (%)
Control	Untreated (*n* = 11)	417 ± 8	5.3 ± 0.2	1.5 ± 0.0	2.5 ± 0.3	44 ± 0
	DL-sulforaphane (*n* = 9)	455 ± 17	4.9 ± 0.2	1.7 ± 0.0	3.4 ± 0.6	43 ± 1
Diabetes	Untreated (*n* = 9)	361 ± 10	23.1 ± 1.1#	2.3 ± 0.1#	41.5 ± 8.0#	46 ± 1#
	DL-sulforaphane (*n* = 11)	372 ± 14	22.0 ± 1.1#	2.3 ± 0.1#	29.5 ± 5.7#	45 ± 1#

# *P* < 0.05 compared to corresponding control group.

**Table 2:** Respiratory control ratio (RCR), respiration in the presence of substrates pyruvate (P) and malate (M) but not adenosine diphosphate (ADP) (state 2 PM), respiration in the presence of substrates pyruvate, malate, and ADP (state 3 PM), respiration in the presence of substrates pyruvate, malate, succinate (S), and ADP (state 3 PMS), respiration in the presence of substrates pyruvate, malate, and ATP-synthase inhibitor oligomycin (Leak respiration), change in respiration after the addition of uncoupling protein (UCP) inhibitor guanosine diphosphate (GDP) (Leak: UCP-mediated), and change in respiration after the addition of adenine nucleotide translocase (ANT) inhibitor CAT (Leak: ANT-mediated) in untreated and DL-sulforaphane treated control or streptozotocin-induced diabetic rats.

Groups	Treatment	RCR	State 2 PM (pmol/s/µg)	State 3 PM (pmol/s/µg)	State 3 PMS (pmol/s/µg)	Leak respiration (pmol/s/µg)	Leak: UCP-mediated (pmol/s/µg)	Leak: ANT-mediated (pmol/s/µg)
Control	Untreated	7.38 ± 0.26	0.39 ± 0.02	2.88 ± 0.20	5.04 ± 0.38	0.28 ± 0.01	0.006 ± 0.003	0.03 ± 0.00
	DL-sulforaphane	7.39 ± 0.16	0.38 ± 0.04	2.76 ± 0.28	5.10 ± 0.41	0.24 ± 0.02	0.000 ± 0.002	0.03 ± 0.00
Diabetes	Untreated	8.19 ± 0.41	0.39 ± 0.02	3.13 ± 0.11	5.67 ± 0.12	0.27 ± 0.01	0.004 ± 0.002	0.03 ± 0.00
	DL-sulforaphane	8.29 ± 0.51	0.34 ± 0.02	2.80 ± 0.09	5.29 ± 0.25	0.25 ± 0.01	0.002 ± 0.002	0.03 ± 0.00

## Discussion

In the present study, the hypothesis tested was that the activation of the Nrf-2 pathway improves kidney oxygen handling in diabetes. The main conclusions are that streptozotocin-induced diabetes results in an increased transport-dependent workload due to increased GFR, which results in an increased QO_2_ but also increased transport-independent QO_2_, evident as decreased transport efficiency (T_Na+_/QO_2_). This ultimately results in decreased cortical pO_2_ in diabetics. Chronic DL-sulforaphane treatment for 4 weeks decreased GFR and subsequently QO_2_, resulting in increased cortical pO_2_ in diabetics. However, transport efficiency was not significantly changed by chronic DL-sulforaphane treatment. Unchanged transport efficiency is supported by data from isolated mitochondria, where oxygen consumption not related to ATP production was not affected by chronic DL-sulforaphane treatment.

DKD is a major cause for end-stage renal disease. No treatment options are available to reverse kidney function, and the medical treatment goal is to preserve GFR. The exact underlying mechanisms for the progression of DKD are not fully understood but involve several pathways. However, a central chain of events where kidney oxygen metabolism is altered, ultimately leading to kidney tissue hypoxia, is important (15). Kidney tissue hypoxia in diabetes can mechanistically be explained by several events occurring in parallel. First, diabetes-induced glomerular hyperfiltration will increase tubular load, hence, tubular transport requiring oxygen. Second, diabetes results in decreased efficiency, meaning that an increased amount of oxygen is required to sustain ATP production. This mechanism is partly mediated by the upregulation and activation of mitochondrial uncoupling via UCP and the ANT. Both these proteins release protons across the inner mitochondrial membrane independently of the ATP-synthase, bypassing ATP production. These mechanisms can regulate mitochondrial membrane potential and subsequently superoxide production. Importantly, UCP is directly activated by superoxide and, therefore, functions as a feedback mechanism to regulate mitochondrial ROS production (5). UCP activity is increased in diabetes due to the hyperglycemia-induced increase in ROS production (3, 4). Nrf2 activation will induce transcription of enzymes involved in degradation of ROS. Consequently, the kidney will work at reduced efficiency, contributing to diabetes-induced kidney hypoxia.

In the present study, we confirmed previous studies (2, 16) that streptozotocin-induced diabetes results in increased kidney QO_2_, decreased transport efficiency (T_Na_/QO_2_), and, as a consequence, decreased intra-renal pO_2_. Inducing Nrf2 by DL-sulforaphane could decrease ROS load in diabetes and therefore preserve oxygen usage efficiency. Furthermore, despite changes in oxygen metabolism *in vivo*, the present study could not confirm a significant change in mitochondrial function or efficiency. Instead, DL-sulforaphane treatment mediates normalization of *in vivo* kidney oxygen handling by decreasing GFR and, hence, tubular workload in terms of T_Na_ resulting in decreased *in vivo* QO_2_ and improved cortical pO_2_. Kidney pO_2_ is important for long-term integrity of the tubular cells. In the present study, diabetes duration and DL-sulforaphane treatment period were 4 weeks. Indeed, animals presented with increased urinary protein excretion, suggesting some degree of glomerular or tubular damage. DL-sulforaphane treatment did not significantly decrease urinary protein excretion, and a longer period of time with DL-sulforaphane treatment could be required for the effect of treatment to be significant.

In conclusion, DL-sulforaphane treatment to induce Nrf2 signaling in the diabetic kidney has hemodynamic effects, reducing GFR and sodium load to the kidney. Hence, it decreases renal QO_2_ and prevents diabetes-induced decreases in cortical pO_2_.
